# Can integrating religiosity and spirituality into postpartum care improve the quality of life in women with preeclampsia

**DOI:** 10.3389/fpsyt.2023.985428

**Published:** 2023-04-27

**Authors:** Robab Rasouli, Azam Maleki, Saeedeh Zenoozian

**Affiliations:** ^1^School of Nursing and Midwifery, Zanjan University of Medical Sciences, Zanjan, Iran; ^2^Social Determinants of Health Research Center, Zanjan University of Medical Sciences, Zanjan, Iran; ^3^Department of Clinical Psychology, School of Medicine, Dr Beheshti Hospital, Zanjan University of Medical Sciences, Zanjan, Iran

**Keywords:** women’s health, preeclampsia, spirituality, quality of life, counseling

## Abstract

**Background:**

Women with a history of preeclampsia frequently have a lower level of physical well-being and emotional problems.

**Objective:**

This study aimed to determine the effect of integrating religiosity and spirituality into postpartum care can improve the quality of life in women with preeclampsia.

**Methods:**

This study was a randomized controlled clinical trial conducted on 40 women with preeclampsia. All eligible participants were allocated to two control and intervention groups using a random blocking method. Data were collected using Mother-Generated Index (MGI) in pre-intervention and 6 weeks later and analyzed using descriptive statistics, Chi-square test, and independent *t*-tests. The significance level was *p* < 0.05.

**Results:**

The mean, Standard deviation (SD) of the total score of MGI before intervention in the intervention group was 5.35 (1.09) which increased to 8.00 (0.50) 6 weeks after intervention. In the control group, the pre-test score of MGI was 5.81 (0.97) which increased to 6.69 (1.37) after 6 weeks of follow-up. The difference between the two groups was statistically significant after the intervention based on an independent *t*-test (*p* = 0.001).

The mean (SD) of five subscales included Feelings toward herself, Feelings toward the child, Feelings toward her husband and others, Feelings toward sex, and Physical health status after intervention in the intervention group statistically significantly increased compared to the control group (*p* < 0.011).

**Conclusion:**

The integration of spiritual counseling with the educational content of postpartum care had a positive impact on improving the postpartum QoL of women with preeclampsia. For better conclusions, a study with a large sample size needed to be conducted in the future.

**Clinical Trial Registration:**

https://en.irct.ir/user/trial/50832/view, identifier IRCT20150731023423N16.

## Introduction

Preeclampsia is one of the hypertensive disorders in pregnancy that affects 4.6% of pregnancies worldwide (1). Also, it is responsible for 10% of preterm births and 3% of perinatal mortality (2). Women with a history of preeclampsia frequently have a lower level of physical well-being (3) and emotional problems such as Post-traumatic stress disorder (PTSD) symptoms compared to women who have had uncomplicated pregnancies, especially if the disease occurs in the early stage of pregnancy (4). Taking care of the mother’s health during pregnancy and preventing her worries, anxieties, and psychological pressures will reduce the severe impairment during this period (5).

According to the World Health Organization definition, quality of life (QoL) is an individual’s understanding of their position in life, goals, expectations, relationships, needs, and beliefs which are affected by one’s cultural context and value systems (6). Spirituality is a significant dimension of quality of life that empowers people to deal emotionally with aspects of illness, life concerns, beliefs, and spiritual values (7). By targeting individual beliefs, people may be able to better handle negative situations and gain greater control than they have at present (8).

Spirituality is distinct from religiosity and is defined as the way people seek and express meaning and purpose in life, and experience their relationship with others, nature, and sacred things. This definition serves as the foundation for the spiritual care approach (9).

Positive effects of spiritual care through the improvement of meaningful communication and positive thinking in controlling postpartum stress disorder among preeclamptic women have been reported (10). Also, spiritual counseling combined with other counseling approaches was found to be significantly effective in reducing anxiety and improving the quality of life of hypertensive patients (11). However, there is a knowledge gap regarding the effectiveness of this approach in improving the quality of life after childbirth in women with preeclampsia. It appears that “spiritual care, “with its broad range of dimensions, as one of the fundamental aspects of holistic treatment in mothers with preeclampsia, could be somewhat effective in controlling their problems and improving their patients’ quality of life. Consequently, the present study examined the effect of integrating religiosity and spirituality into postpartum care on the quality of life in women with preeclampsia.

## Hypothesis

Integrating religiosity and spirituality into postpartum care is effective in improving the quality of life among women with preeclampsia compared to the control group.

## Materials and methods

### Study design

This study was a randomized controlled trial that was conducted on 40 women with preeclampsia who were admitted to the postpartum ward at Ayatollah Mousavi Teaching Hospital in Zanajn a city in northwest Iran from October 2020 to May 2021.

### Participants’ inclusion and exclusion criteria

**Figure 1:**
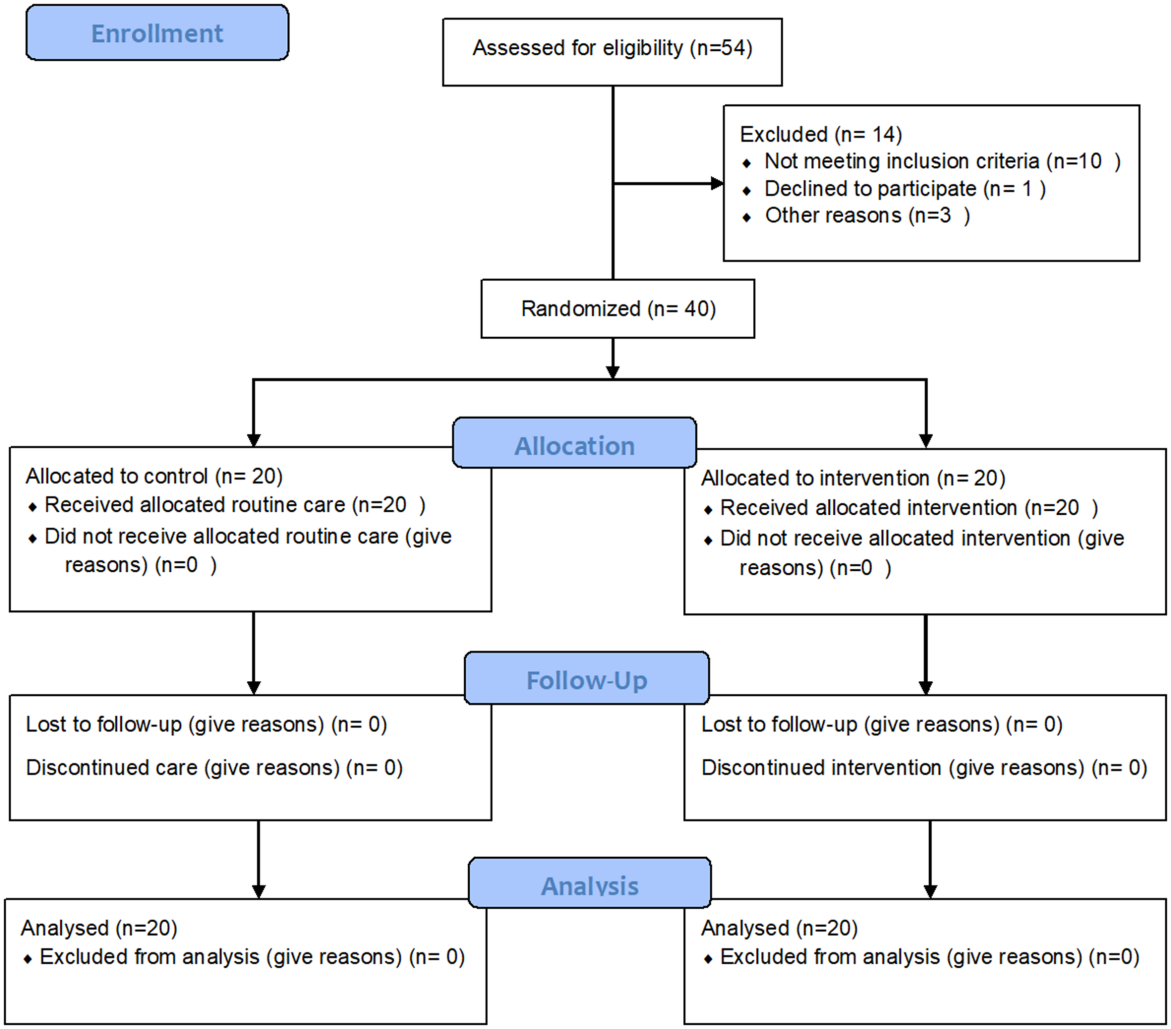
The process of sampling.

Among 54 preeclamptic women who were evaluated by the researcher, 40 women met the inclusion criteria and 14 women were excluded before being randomly allocated to groups. Preeclampsia was diagnosed based on the International Society for the Study of Hypertension in Pregnancy (ISSHP) guidelines (12). After the interventions, there was no attrition in our study. The process of sampling showed in Figure 1.

Inclusion criteria included hospitalization with a diagnosis of preeclampsia less than 5 days, willingness to participate in the study, having a delivery with a singleton pregnancy, having a minimum of elementary education, getting a score less than 22 based on the General Health Questionnaire (GHQ-28), and mild to moderate level of Daily Spiritual Experiences Scale (DSES). Exclusion criteria included having a history of chronic diseases, having a known mental illness, use of any psychiatric medicine, a known neonatal abnormality, intrauterine fetal death, having postpartum complications (such as bleeding and infection), no phone number for calling, and changing the treatment process (for example, transfer to the intensive care unit). The sample size was calculated based on the quality-of-life variable reported by Kalhornia’s study (11). Accordingly, the mean (SD) of quality of life was $M_{2=43.52}$, $S_{2=13.70}^{2}$ in intervention group, and $M_{1=36.6}$, $S_{1=25.79}^{2}$ in control group, respectively. With considering *α* = 0.05, Power = 95%, and a 15% drop, the sample size was calculated as 20 people in each group.

### Sampling

The researcher referred to the postpartum ward of Ayatollah Mousavi Teaching Hospital in Zanjan and after obtaining written consent to participate in the study from all participants, the inclusion and exclusion criteria were checked and eligible women completed the demographic questions and postnatal quality of life Questionnaire (Mother-Generated Index).

### Randomization

All eligible participants were allocated to two control and intervention groups using a random blocking method with block sizes of four. Randomization was done by a person who was not directly involved in our study using a research Randomizer. It is a free resource and a quick way for researchers to generate random numbers or assign participants to control and intervention groups. The generated numbers were placed in an opaque envelope and then sequentially numbered. When the participants entered the study, the assigned group was identified by opening each envelope.

#### Routine care

All women with preeclampsia were admitted to the postpartum ward at Ayatollah Mousavi Teaching Hospital in Zanajn, in addition, to controlling bleeding, prescribing medicine, and recording vital signs, received an educational package that included personal hygiene, breastfeeding, and warning signs of maternal and neonatal complications. Women are not routinely educated on the content of spirituality postpartum.

#### Intervention

In this study, the intervention group received counseling individually in five sessions with spiritual content based on Richards and Bergin’s protocol by the first author. A spiritual content program was developed by the researchers and a religious counselor. In the counseling group, the intervention was started 12 h after delivery and stabilization of the mother’s general condition. Three sessions were held in the postpartum ward during the hospitalization period in a place with adequate space to observe social distancing and other health protocols to prevent Covid-19 diseases such as proper air conditioning and using a face mask. The next two sessions were held weekly after discharge by telephone counseling. Each counseling session lasted 45–60 min.

Table 1 shows the content of the intervention separately by session. The control group received routine care.

**Table 1:** Spirituality-oriented counseling session protocol.

Sessions	Session contents
First session	Introducing yourself and getting to know the patient, expressing the goals and intervention
	Discussing the preeclampsia, paying attention to the patient’s attitude toward the disease, negative emotions, stress, and worries, and strategies for dealing with them
	Discussing the concept of quality of life, dimensions of quality of life, and the importance of self-awareness
	Discussing one’s expectations of life, community, values, one’s attitude toward spiritual matters, the role of God and spirituality in her life, the patient’s positive experiences of using spiritual matters to deal with stressful situations in her life, recalling the role of women in creation, spiritual aspects of pregnancy and childbirth, maternal role from an Islamic perspective
Second session	Reviewing the previous session’s content, reminding mothers of their responsibility from the perspective of the Qur’an and the spiritual reward of mothers
	Teaching relaxation with breathing exercises and repeating them 10 to 15 times a day
	Recommend listening to nature music and the sound of the Qur’an during the day
	Recommend repeated relaxation with breathing exercises in the following days
Third session	Reminiscing about previous sessions, assessing one’s mood, attitude, and beliefs about life, the role of thanking God in enduring hardships, increasing hope and self-confidence in adapting and accepting situations, the role of reading the Bible in patients’ comfort, and religious people’s stories
	Encouraging patients to talk to people who feel positive about them
	Increasing intimacy with the spouse by involving them in life responsibilities
Fourth session	Trust, empathy, and honesty between midwives and mothers to properly communicate during sessions, listen carefully to the physical and mental worries and concerns of patients’ fears, provide psychological support to patients, strengthen people’s inner hope and strength, and use positive sentences
	By energizing and strengthening healthy and constructive thoughts, assisting the patient in finding meaning in life, and understanding that none of life’s events is beyond fate, one who believes in God to the entire world can be saved from feelings of pessimism, emptiness, and despair
	Encourage patients to provide the necessary facilities for religious affairs, encourage patients to read Holy Scripture, and encourage patients to refer to people with whom they feel at ease
	Assure the patient that the midwife is always available for psychological support to her clients
	Have forgiveness in your life and let go of your rage toward the guilty person and sinner
	Encourage patients to donate to charity
Fifth session	Repetition of the fourth session

### Data collection tools

The outcome of the study was to determine the postnatal quality of life.

Data collection tools included demographic questions, postnatal quality of life Questionnaire (Mother-Generated Index) that were completed in two-stage (pre-intervention and 6 weeks after the last session of counseling).

The questionnaire included education, spouse education, family income, insurance status, job, spouse job, residence, homeownership, and parity.

### Postnatal quality of life questionnaire: mother-generated index

The MGI was designed by Symon et al. It is a subjective tool to measure the postnatal quality of life and contains 30 questions in eight areas. The dimensions of the questionnaire include the mother’s feelings toward herself (6 questions), feelings toward the child (3 questions), feelings toward her husband and other family members (8 questions), feelings toward sex with a spouse (3 questions), physical health status (7 questions), Satisfaction with childbirth (one question), the status of the economics associated with childbirth (one question), and the re-selection of the method of childbirth in subsequent pregnancies (one question). The MGI score was calculated by weighting the means of the values on visual analog scales between one and 10. A high score in any area indicates a better situation in terms of quality of life. The face, criterion, and construct validity of the questionnaire were at an acceptable level (13). Psychometric properties of the Persian version of the questionnaire were tested by Khabiri, et al. In their study, the face, criterion, and construct validity of the questionnaire were at an acceptable level (14). The reliability of the questionnaire in the present study was confirmed by calculating Cronbach’s alpha coefficient of 0.78.

### Data analysis

Data were analyzed using SPSS 16. Descriptive statistics were used to describe the demographic information. The Chi-square test was used to compare demographic characteristics between the two groups. Kolmogorov–Smirnov test showed that MGI scores have a normal distribution. Therefore, to compare the scores of MGI between groups independent *t*-tests were used. The significance level was *p* < 0.05.

### Results

Among 54 women evaluated by the researcher, the data of 40 women met the eligibility criteria for analysis.

### Demographic characteristic

In both groups, the most of participants have Elementary & middle school level education (55%). Regarding the parity, the most of participants in the control group had >1 parity (65%), and in the intervention group was equal to one parity (60%). Regarding the family-income and insurance, and job status, the most of participants in both groups were at enough level, had insurance, and had jobs were housewives. Also, personal property was the most common form of homeownership in both groups. The residence of most people was rural in the intervention group (55%) and urban (65%) in the control group. Regarding their spouses’ jobs and education, most of them in both groups have Elementary and middle school level and self-employed jobs. The comparison of demographic characteristics using the Chi-square showed that there is no significant difference between the two groups (Table 2).

**Table 2:** The comparison of participants’ demographic characteristics between two groups.

Demographic Characteristics		Groups				*p*-value
		Control		Intervention		
		Frequency	Percentage	Frequency	Percentage	
Education	Elementary and middle school	11	55.0	11	55.0	0.368
	High school& diploma	6	30.0	3	15.0	
	Academic education	3	15.0	6	30.0	
Parity	1	7	35.0	12	60.0	0.113
	>1	13	65.0	8	40.0	
Job	Employed	4	20.0	6	30.0	0.465
	Housewife	16	80.0	14	70.0	
Residency	Urban	7	35.0	11	55.0	0.204
	Rural	13	65.0	9	45.0	
Homeownership	Personal property	16	80.0	11	55.0	0.239
	Rent	3	15.0	7	35.0	
	With family	1	5.0	2	10.0	
Insurance status	Yes	12	60.0	15	75.0	0.311
	No	8	40.0	5	25.0	
Family Income	Enough	14	70.0	13	65.0	0.938
	Somehow enough	5	25.0	6	30.0	
	Insufficient	1	5.0	1	5.0	
Spouse Education	Elementary-middle school	11	55.0	12	60.0	0.749
	High school diploma	7	35.0	5	25.0	
	University	2	10.0	3	15.0	
Spouse job	Government-Employees	2	10.0	2	10.0	1.000
	Self-employed	18	90.0	18	90.0	

### The mother-generated index

The mean, Standard deviation (SD) of the total score of MGI before intervention in the intervention group was 5.35 (1.09) which increased to 8.00 (0.50) 6 weeks after intervention. In the control group, the pre-test score was 5.81 (0.97) which increased to 6.69 (1.37) after 6 weeks of follow-up. The difference between the two groups was statistically significant after 6 weeks of follow-up based on an independent *t*-test (*p* = 0.001).

The mean (SD) of five subscales included Feelings toward herself, Feelings toward the child, Feelings toward her husband and others, Feelings toward sex, and Physical health status after intervention in the intervention group statistically significantly increased compared to the control group (*p* < 0.011). But three subscales included satisfaction with childbirth, the economic difficulty, and the re-selection of the method of childbirth after intervention based on an independent *t*-test was not significant (*p* > 0.05; Table 3).

**Table 3:** The comparison of MGI scores between two groups in pre-intervention and 6 weeks after intervention.

MGI	Group	Pre-test		*p* *value	Post-test		*p* *value
		Mean	SD		Mean	SD	
Feelings toward herself	Intervention	5.29	1.63	0.580	7.47	1.18	0.001
	Control	5.54	1.15		5.60	1.37	
Feelings toward the child	Intervention	6.33	1.75	0.677	8.41	1.38	0.001
	Control	6.55	1.49		7.00	1.05	
Feelings toward her husband and other	Intervention	5.56	1.43	0.047	8.52	0.740	0.001
	Control	6.50	1.45		6.97	0.713	
Feelings toward sex	Intervention	3.45	1.02	0.631	6.16	1.00	0.011
	Control	3.63	1.35		5.25	1.16	
Physical health status	Intervention	5.65	1.53	0.543	8.35	0.89	0.011
	Control	5.93	1.41		7.50	1.11	
Satisfaction with childbirth	Intervention	5.40	2.28	0.639	8.70	1.83	0.072
	Control	5.75	2.40		7.65	1.75	
The economics difficulty	Intervention	4.90	2.02	0.252	8.25	1.77	0.064
	Control	5.70	2.31		7.30	1.34	
The re-selection of the method of childbirth	Intervention	5.10	1.83	0.361	7.90	1.77	0.119
	Control	5.65	1.92		7.10	1.37	
Total score	Intervention	5.35	1.09	0.166	8.00	0.50	0.001
	Control	5.81	0.97		6.69	0.79	

* Independent *t*-test, Mother-Generated Index (MGI), Standard deviation (SD).

## Discussion

The current study was conducted to examine the effect of integrating spirituality into postpartum care on improving the quality of life in women with preeclampsia. Our result revealed that the intervention can improve the total score of quality of life and its subscales compared to the control group. According to our knowledge, this is the first study to assess the effect of integrating spirituality into postpartum care on the quality of life among women with preeclampsia. However, our results are consistent with studies that were conducted on other high-risk pregnancy samples. In this regard, the result of two similar studies showed that the implementation of Islamic- spiritual psychotherapy could be improved the quality of life of infertile women (15, 16). Also, integrating cognitive-behavioral therapy with Islamic spirituality instructions had an effective impact on the quality of life of pregnant women (17). Beigi et al. showed that the implementation of group spiritual therapy was effective in reducing anxiety and increasing the quality-of-life score in women with gestational diabetes (18). Also, similar efficacy has been reported in another study conducted by Niaz Azari et al. in women with gestational diabetes (19). Constituency in results emphasized that the spiritual-based approach can be used to improve the quality of life of women in the antenatal and postpartum periods.

The positive effects of spiritual counseling on improving blood pressure, life satisfaction, and quality of life of non-pregnant hypertensive patients have also been emphasized in some studies (11, 20). Religion and spirituality can increase QoL by changing people’s attitudes, increasing their sense of responsibility toward themselves and others, promoting the search for meaning in life, and having a greater sense of happiness and self-esteem. Spiritual experience is a unique experience that entails understanding the meaning of life, having positive experiences in life, and feeling happy, and satisfied with life (21).

In the current study, intervention can improve the sense of women toward themselves, child, their husbands, and others. Participants had positive feelings toward sex and Physical health status after the intervention. While, satisfaction with childbirth, the economic difficulty, and the re-selection of the delivery method have not major changes. Similar to our result, the positive effect of integration of spirituality in antenatal care can improve the general health perceptions and physical and emotional role function of healthy pregnant women (22). On the contrary Dehestani et al. revealed that spiritual health had a positive effect on preventing labor pains and childbirth satisfaction in women (23). Furthermore, a negative correlation between spiritual intelligence and fear of giving birth was reported (24). The reason for inconsistent results regarding satisfaction with childbirth could be that in our study the intervention was performed after delivery and in mothers with high-risk pregnancies.

### Strength of study

All the principles of control trial studies were observed in this study and we do not have a loss of following in participants. Data collection tools were standard and psychometric properties of the Persian form of the questionnaires have been evaluated based on Iranian culture.

## Limitation

The sample size was small and the follow-up period was short. Also, samples were limited to the participants of the Shiite religion, which can affect the generalizability of findings. It is suggested that a similar study should be performed on other ethnicities and cultures in future studies. Furthermore, for better conclusions about the long-term effects of the spiritual-based intervention on postpartum QoL, studies with a long follow-up period and large sample size are needed to be conducted in the future.

### Highlighted

Preeclampsia can affect the physical and emotional well-being of women.

Spiritual counseling can improve the postpartum QoL of women with preeclampsia compared to the control group.

## Conclusion

Preliminary results show spiritual counseling had a positive impact on improving the postpartum QoL of women with preeclampsia. For better conclusions, a study with a large sample size needed to be conducted in the future.

## Data availability statement

The original contributions presented in the study are included in the article/Supplementary materials, further inquiries can be directed to the corresponding author.

## Ethics statement

The studies involving human participants were reviewed and approved by Zanjan University of Medical Sciences, Iran, with the approval number IR.ZUMS.REC.1398.376. The patients/participants provided their written informed consent to participate in this study.

## Author contributions

RR undertaken the conception, design of the study, and data collection process. AM was the supervisor who also contributed to the design of the study and reporting of the results, supervised analysis, interpretation, and reporting. SZ as the second supervisor contributed to all the stages of the study. All authors contributed to the drafting and revising of the article and agreed with the final version of the manuscript to be submitted to the journal, they also meet the criteria of authorship.

## Funding

This study was part of MSc thesis and funded by the Research Deputy of Zanjan University of Medical Sciences, Iran, with the approval number (Code “A-11-344-13”).

## Conflict of interest

The authors declare that the research was conducted in the absence of any commercial or financial relationships that could be construed as a potential conflict of interest.

## Publisher’s note

All claims expressed in this article are solely those of the authors and do not necessarily represent those of their affiliated organizations, or those of the publisher, the editors and the reviewers. Any product that may be evaluated in this article, or claim that may be made by its manufacturer, is not guaranteed or endorsed by the publisher.

## Abbreviations

PTSD: Post-traumatic stress disorder
SD: Standard deviation
MGI: Mother-Generated Index
QoL: The Quality of Life
COVID-19: Coronavirus disease
GHQ-28: The General Health Questionnaire
DSES: Daily Spiritual Experiences Scale
ISSHP: Hypertension in Pregnancy

## References

[1] Abalos E Cuesta C Grosso AL Chou D Say L. Global and regional estimates of preeclampsia and eclampsia: a systematic review. Eur J Obstet Gynecol Reprod Biol. (2013) 170:1–7. doi: 10.1016/j.ejogrb.2013.05.005, PMID: 23746796

[2] Bilano VL Ota E Ganchimeg T Mori R Souza JP. Risk factors of pre-eclampsia/eclampsia and its adverse outcomes in low-and middle-income countries: a WHO secondary analysis. PLoS One. (2014) 9:e91198. doi: 10.1371/journal.pone.0091198, PMID: 24657964 PMC3962376

[3] Roes EM Raijmakers MT Schoonenberg M Wanner N Peters WH Steegers EA. Physical well-being in women with a history of severe preeclampsia. J Matern Fetal Neonatal Med. (2005) 18:39–45. doi: 10.1080/14767050500127740 16105790

[4] Roberts L Davis GK Homer CS. Depression, anxiety, and post-traumatic stress disorder following a hypertensive disorder of pregnancy: a narrative literature review. Front Cardiovasc Med. (2019) 6:147. doi: 10.3389/fcvm.2019.00147, PMID: 31649935 PMC6794436

[5] Golshani F Gholmohammad Nejad GR Aghdasi AN. The effectiveness of teaching life skills during pregnancy on pregnant mothers’ mental health. J Instr Eval. (2014) 7:89–101.

[6] Group W. The World Health Organization quality of life assessment (WHOQOL): position paper from the World Health Organization. Soc Sci Med. (1995) 41:1403–9. doi: 10.1016/0277-9536(95)00112-K 8560308

[7] Sotodeh-Asl N Neshat-Dust H Kalantari M Talebi H Khosravi A. Comparison of effectiveness of two methods of hope therapy and drug therapy on the quality of life in the patients with essential hypertension. J Clin Psychol. (2010) 2:1–5.

[8] Yilmaz CK Kara FŞ. The effect of spiritual well-being on adaptation to chronic illness among people with chronic illnesses. Perspect Psychiatr Care. (2021) 57:318–25. doi: 10.1111/ppc.12566, PMID: 32596844

[9] Richards PS Bergin AE. Religious and spiritual assessment. Richards PS Bergin AE. A spiritual strategy for counseling and psychotherapy. Washington, DC: American Psychological Association. (2005).

[10] Kamali Z Tafazoli M Ebrahimi M Hosseini M Saki A Fayyazi-Bordbar MR. Effect of spiritual care education on postpartum stress disorder in women with preeclampsia. J Educ Health Promot. (2018):7. doi: 10.4103/jehp.jehp_170_17 29963566 PMC6009134

[11] Kalhornia-Golkar M Banijamali S Bahrami H Hatami H Ahadi H. Effectiveness of mixed therapy of stress management training and spiritual therapy on level of blood pressure, anxiety and quality of life of high blood pressure patients. J Clin Psychol. (2014) 6:1–11.

[12] Tranquilli A Dekker G Magee L Roberts J Sibai B Steyn W. The classification, diagnosis and management of the hypertensive disorders of pregnancy: A revised statement from the ISSHP. Pregnancy Hypertens. (2014) 4:97–104. doi: 10.1016/j.preghy.2014.02.001 26104417

[13] Symon A McGreavey J Picken C. Postnatal quality of life assessment: validation of the mother-generated index. BJOG Int J Obstet Gynaecol. (2003) 110:865–8. doi: 10.1111/j.1471-0528.2003.02030.x, PMID: 14511971

[14] Khabiri R Rashidian A Montazeri A Symon A Foroushani AR Arab M. Validation of the mother-generated index in Iran: a specific postnatal quality-of-life instrument. Int J Prev Med. (2013) 4:1371–9. PMID: 24498492 PMC3898442

[15] Moazedi K Porzoor P Pirani Z Adl H Ahmadi H. The effectiveness of Islamic teaching based religious-spiritual psychotherapy on quality of life, in infertile women. J Health. (2018) 9:589–98.

[16] Pirani Z. The effectiveness of Islamic teaching based religious-spiritual psychotherapy on quality of life, in infertile women. Iran: University of Mohaghegh Ardabili (2019).

[17] Zamani SN Zarei E Alizadeh KH Naami AZ. Effectiveness of combination of cognitive-behavioral therapy and resilience training based on Islamic spirituality and cognitive flexibility on postpartum depression, fear of labor pain and quality of life. Hormozgan Med J. (2019) 22:e86489. doi: 10.5812/hmj.86489

[18] Beigi A Habibi S Rezaei Hesar H Niasty R Shams Ali Z Ashoori J. Effect of spiritual training on decreased anxiety and increased quality of life of women with gestational diabetesin the assement of nursing and modern care. J Diabetes Nurs. (2016) 4:19–29.

[19] Niaz Azari M Abdollahi M Zabihi Hesari NK Ashoori J. Effect of spiritual group therapy on anxiety and quality of life among gestational diabetic females. J Relig Health. (2017) 5:11–20.

[20] Moeini M. The effect of an Islamic spiritual program on life satisfaction of elderly patients with hypertension. J Clin Nurs Midwifery. (2016) 4:93–103.

[21] Lent RW. Toward a unifying theoretical and practical perspective on well-being and psychosocial adjustment. J Couns Psychol. (2004) 51:482–509. doi: 10.1037/0022-0167.51.4.482

[22] MonfaredKashki M Maleki A Amini K. The effect of integrating midwifery counseling with a spiritual content on improving the antenatal quality of life: a randomized controlled trials. J Mother Child. (2021) 26:18–26. doi: 10.34763/jmotherandchild.20222601.d-22-00003 PMC10062411 35853465

[23] Dehestani H Tayebi N Moshfeghy Z Akbarzadeh M. The role of spiritual health experience with intensity and duration of labor pain while childbearing and postpartum. Curr Womens Health Rev. (2022) 18:77–84. doi: 10.2174/1573404817666210310165046

[24] Abdollahpour S Khosravi A. Relationship between spiritual intelligence with happiness and fear of childbirth in Iranian pregnant women. Iran J Nurs Midwifery Res. (2018) 23:45–50. doi: 10.4103/ijnmr.IJNMR_39_16, PMID: 29344046 PMC5769185

